# Supraclavicular ultrasound-guided subclavian vein cannulation in infants under 5 kg

**DOI:** 10.1186/cc10814

**Published:** 2012-03-20

**Authors:** P Kenderessy

**Affiliations:** 1 Faculty Children Hospital, Banska Bystrica, Slovakia

## Introduction

Central venous cannulation is at some point difficult in small children and is associated with many complications especially in multiple-attempt cases. Various techniques exist to achieve successful cannulation. Ultrasound (US)-guided techniques are reported to be safe and reduce the rate of complications for internal jugular vein (IJV) cannulation. We describe an US-guided supraclavicular approach to another central vein - the subclavian vein (SCV). The supraclavicular approach to the SCV with anatomical landmarks was described by Yoffa, but physicians are hesitant to use this technique because of the short distance to pleura.

## Methods

The principle of the US-navigated technique is to find the SCV at the supraclavicular level and to obtain a longitudinal view of the SCV and to allow access to the vein in-plane view (absolute control of the needle). The ultrasound probe (2.5 cm, 6 to 13 MHz) was placed above the clavicle to visualize the IJV and tilted showing the subclavian artery and SCV in longitudinal view. This view permitted an in-plane puncture of the vein avoiding arterial or plural hit.

## Results

Seventy-eight infant and newborns under 5 kg (1.2 to 5 kg) and 83 SCV cannulations were enrolled in this observational study during a period of 11 months (January 2011 to November 2011). All cannulations were performed by a single anesthesiologist trained for ultrasound in central line cannulation with established eye-hand coordination (5 years experience with peripheral blocks under US). For all cases the SCV was easily and quickly visualized, one case had an extremely narrow SCV. The US window for cannulation was always established for free in-plane placement of the needle. The overall success rate for puncture was 100% and for cannulation was 98%. In the case with an extremely narrow vein (because of oedema and stricture) the SCV was punctured but it was impossible to pass the catheter in. The success rate of puncture at first attempt was 97%, at second attempt was 100%. A second attempt was necessary in two cases because needle visualization and angle of the needle movement were not considered correct. No complication was reported.

## Conclusion

A supraclavicular US-guided approach to SCV cannulation is safe and effective possibility for central vein cannulation in small infants. More studies are needed to establish a learning curve for pure paediatric intensivists without experience with US navigation.

